# Direct and Indirect Effects of Floral Defoliation on Photochemical and Non‐Photochemical Chlorophyll Fluorescence Dynamics of a Semiarid Bunchgrass

**DOI:** 10.1002/pei3.70119

**Published:** 2026-02-19

**Authors:** Erik P. Hamerlynck, Rory C. O'Connor

**Affiliations:** ^1^ USDA Agricultural Research Service Research Ecologist Eastern Oregon Agricultural Research Center Burns Oregon USA

**Keywords:** *Agropyron cristatum*, *F*_v_/*F*_m_, herbivory, parental provisioning, photoprotection, photosynthesis, PSII quantum efficiency, reproduction

## Abstract

Photosynthetic florets support reproductive development and energetic seed provisioning of semi‐arid bunchgrasses whose population dynamics rely mainly on sexually produced propagules. Photosynthetic gas exchange studies including crested wheatgrass ( *Agropyron cristatum* ) have found compensatory increases in seed‐head photosynthesis following floral defoliation are accompanied by reduced light‐adapted PSII quantum yield (*ϕ*_PSII_). We undertook a field experiment to ascertain if altered *ϕ*_PSII_ and optimal PSII quantum yield (*F*_v_/*F*_m_) were concurrent with higher quantum yield of regulated (*ϕ*_NPQ_) or unregulated (*ϕ*_NO_) non‐photochemical PSII absorbed energy dissipation. We quantified these responses in directly affected basal florets, and in unclipped distal florets to establish indirect responses to tissue loss. Clipping basal florets reduced *F*_v_/*F*_m_ and increased *ϕ*_NPQ_, indicating effective engagement of regulated non‐photochemical photoprotection, but did not reduce *ϕ*_PSII_ compared to unclipped controls. Florets distal to clipped basal florets had higher *ϕ*_PSII_ but not *F*_v_/*F*_m_, with concurrently lower *ϕ*_NO_ compared to those distal to unclipped controls, possibly due to improved electron transport due to carbon supplementation from damaged basal florets to developing distal propagules. These results demonstrate crested wheatgrass possesses a remarkably integrated reproductive photosynthetic apparatus, facilitating its ability to consistently produce viable seed cohorts under conditions that limit native bunchgrasses reproductive success.

## Graphical abstract

After herbivory, crested wheatgrass florets must scrub excess absorbed light as heat via regulated physiological processes or less effective unregulated means. We found clipped florets heightened physiological control while undamaged florets reduced unregulated dissipation of excess light energy.

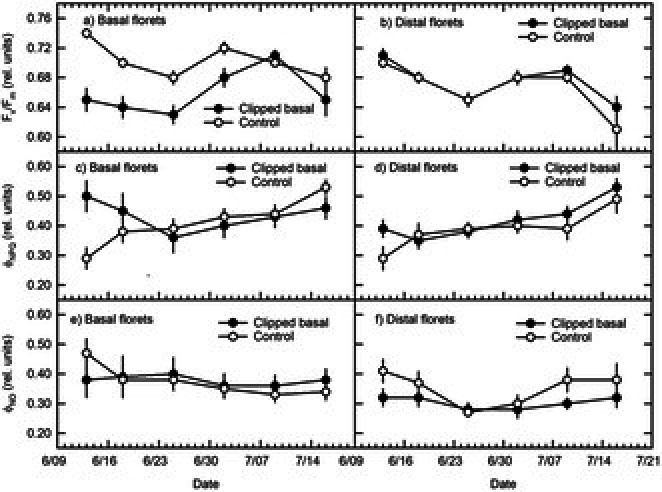

## 1 Introduction

In semiarid sagebrush steppe bunchgrasses, photosynthetic activity in the seed‐head is an important determinant of seed quality, as field experiments have shown seed‐head shading markedly reduces seed specific mass, an indicator of parental energetic provisioning (Hamerlynck and O'Connor 2021, Hamerlynck et al. 2024). This is important as these grasses' population dynamics rely almost exclusively on sexual reproduction (Liston et al. 2003; Hamerlynck and Davies 2019) and seed specific mass is associated with seedling traits that contribute to successful establishment (Quigley et al. 2023). The highly successful exotic Eurasian bunchgrass crested wheatgrass, *Agropyron cristatum* ([L.] Gaertn.), shows compensatory increases in photosynthesis in damaged florets that serve to enhance its reproductive effort in the remaining undamaged florets (Hamerlynck et al. 2023), a result not observed in native bunchgrasses (Quigley et al. 2024). In both these studies, floral defoliation decreased light‐adapted PSII yield (*ϕ*_PSII_; Genty et al. 1989), depending on reproductive phenological stage (Hamerlynck et al. 2023), or genotypic variation associated with selection for high reproductive output (Quigley et al. 2024). Herbivory‐induced alterations to *ϕ*_PSII_ are well‐documented across a wide variety of plants and different modes of herbivory and are often associated with engagement of non‐photochemical photoprotective mechanisms (Nabity et al. 2012; Jänkäpää et al. 2013; Barton 2016; Henschel et al. 2024). While well‐studied in vegetative tissue, photoprotective activity in response to defoliation in graminoid reproductive structures has not been well‐documented.

Non‐photochemical dissipation of excess light energy is a critical eco‐functional feature of all oxygenic‐photosynthetic organisms (Demmig‐Adams and Adams III 1992; Osmond 1994; Derks et al. 2015), and is a function of the quantum yields of physiologically regulated (*ϕ*_NPQ_) and unregulated constituent quenching processes (*ϕ*_NO_; Klughammer and Schreiber 2008), both of which vary in response to environmental conditions and between species with distinct ecological tolerances (Proctor and Smirnoff 2011; Samson et al. 2019). Photosynthetic activity within the seed‐head plays a central role in the energetic provisioning and attendant reproductive effort of aridland bunchgrasses (Hamerlynck and O'Connor 2021; Hamerlynck et al. 2024). Understanding how floret *ϕ*_NPQ_ and *ϕ*_NO_ respond to defoliation may be important, as these may modulate compensatory increases in photosynthesis in damaged florets associated with increased reproductive potential in the remaining unaffected florets (Hamerlynck et al. 2023).

Here, we present an experimental field study assessing the photochemical and non‐photochemical responses of crested wheatgrass to simulated floral herbivory. As stated earlier, crested wheatgrass has demonstrated capacity to increase affected floret photosynthesis in response to defoliation, and that this is accompanied by a marked decrease in *ϕ*_PSII_ (Hamerlynck et al. 2023). In addition to *ϕ*_PSII_, we measured optimal PSII quantum yield (*F*_v_/*F*_m_; Genty et al. 1989) to establish the full photochemical response to defoliation. We expected clipping to induce a decrease in *F*_v_/*F*_m_ and *ϕ*_PSII_ and it would be concurrent with increased *ϕ*_NPQ_, indicating these declines are photoprotective and are under physiological regulation. Moreover, we also tracked *F*_v_/*F*_m_, *ϕ*_PSII_, *ϕ*_NPQ_ and *ϕ*_NO_ in florets distal to clipped and unclipped controls to establish any indirect effects of floral tissue removal on photochemical and non‐photochemical processes.

## 2 Materials and Methods

Field work was performed from June 10 to July 17, 2025, in a level area of intact Wyoming big sagebrush ( *Artemisia tridentata* [Nuttall] spp. *wyomingensis* Beetle & Young) in a stand of naturally established crested wheatgrass on the Northern Great Basin Experimental Range (NGBER; 119.691709 W, 43.472600 N, 1395.4 m ASL), located ca. 70 km from Burns, OR. Supporting environmental measurements of daily average rooting zone volumetric soil moisture (*θ*_soil_) at 10 cm depth were made at a soil moisture monitoring array established in 2018 (see Hamerlynck and Ziegenhagen 2020 for installation and data acquisition details). Precipitation data was obtained from a National Atmospheric Deposition Program/National Trends Network (NADP/NTN) site (Site OR07; https://nadp.slh.wisc.edu/precipitation/) located 206 m SE from the sampling location.

Four days prior to chlorophyll fluorescence measurements, eight reproductive culms with recently emerged seed‐heads were tagged at the base on eight randomly selected plants. Two days later, four of the culms on each plant had ca. 50% of their basal floret area ca. 3 cm from the distal tip of the inflorescence clipped using scissors as per Hamerlynck et al. (2023). Night‐time dark‐adapted (ca. 2:00–4:30 AM, PDT) and mid‐morning light‐adapted (ca. 8:30–10:00 AM PDT) chlorophyll fluorescence (F) measurements were made on basal and distal florets with a Li‐600‐N porometer/fluorometer (LiCOR Instruments, Lincoln, NE). On each of the six seasonal sampling dates, two clipped and two control culms were randomly selected for night‐time measurements from marked culms on each plant (*n* = 16 per treatment type, total *n* = 32 culms for each time point), with their identifying information recorded for subsequent daytime remeasurement. Tissue was enclosed across the full length of a 0.15 × 0.05 cm cuvette and exposed to a modulated F measuring beam provided by 2 LED light sources focused on the cuvette, filtered by a 750 ± 40 nm band‐pass filter, with fluorescence detected from 700 to 780 nm. Determination of baseline fluorescence (F_o_) in the dark and light‐adapted steady‐state fluorescence (F_s_) under incident ambient sunlight were established via a sub‐saturating modulated beam pulsed at 4–8 Hz over 2–5 s. This was followed by a saturating flash of 10,000 μmol m^−2^ s^−1^ intensity with a pulse width of 667 ns and 250–750 kHz frequency to determine dark‐adapted maximum fluorescence (F_m_) and light‐adapted maximum fluorescence yield (F_m_′). F_s_ and F_m_′ were determined using incident photosynthetic photon flux density (PPFD in μmol m^−2^ s^−1^) measured concurrently with a Si‐photodiode quantum sensor integrated into the Li‐600‐N, as the actinic light source, with all samples oriented perpendicular to the sun to receive similar levels of incident actinic PPFD. From these, maximum PSII quantum efficiency (*F*_v_/*F*_m_ (*F*_m_ – *F*_o_)/*F*_m_) and light‐adapted PSII quantum yield ((*ϕ*_PSII_; *F*_m_′ – *F*_s_)/*F*_m_′) were determined. *F*_m_ and *F*_m_′ were used to estimate the quantum yield of non‐photochemical energy loss (NPQ) in PS II that is physiologically regulated (*ϕ*_NPQ_; *ϕ*_NPQ_ = (*F*_s_/*F*_m′_) – (*F*_s_/*F*_m_)) and unregulated (*ϕ*_NO_; *ϕ*_NO_ = *F*_s_/*F*_m_) (Klughammer and Schreiber 2008).

Split‐plot repeated measures analysis of variance (RM‐ANOVA) (Statisix v.8.0; Analytical Software, Tallahassee, FL) was used to test for differences in photochemical and non‐photochemical performance of clipped and unclipped basal florets, and intact florets distal to these. Individual culms were considered replicates for our statistical analyses, using data from basal and distal florets from 16 clipped and 16 unclipped culms on each sampling date (total *n* = 32 culms). We did so considering the independent and modular nature of plant functional structure and light interception at a canopy level in perennial bunchgrasses (Sprugel et al. 1991; Ryel et al. 1993). As we were not interested in comparing basal and distal floret performance but rather the effects of basal clipping within these, we ran separate RM‐ANOVA for each position. Whole‐plot between treatment effect was clipping treatment (clipped vs. unclipped control), with the clip‐by‐replicate culm interaction as the whole‐plot F‐test as error term. We grouped the six sampling dates into three phenological stages: pre‐anthesis (June 13 and 18), anthesis (June 25 and July 2), and post‐anthesis (July 9 and 16), and used these three phenological stages as the sub‐plot effect, along with the stage‐by‐clipping interaction, using the stage‐by‐clip‐by‐replicate culm interaction as *F*‐test denominator. *F*‐test results were considered significant at associated *p* < 0.05; with post hoc means comparisons made using α‐adjusted least significant difference (LSD) test.

## 3 Results

**FIGURE 1:**
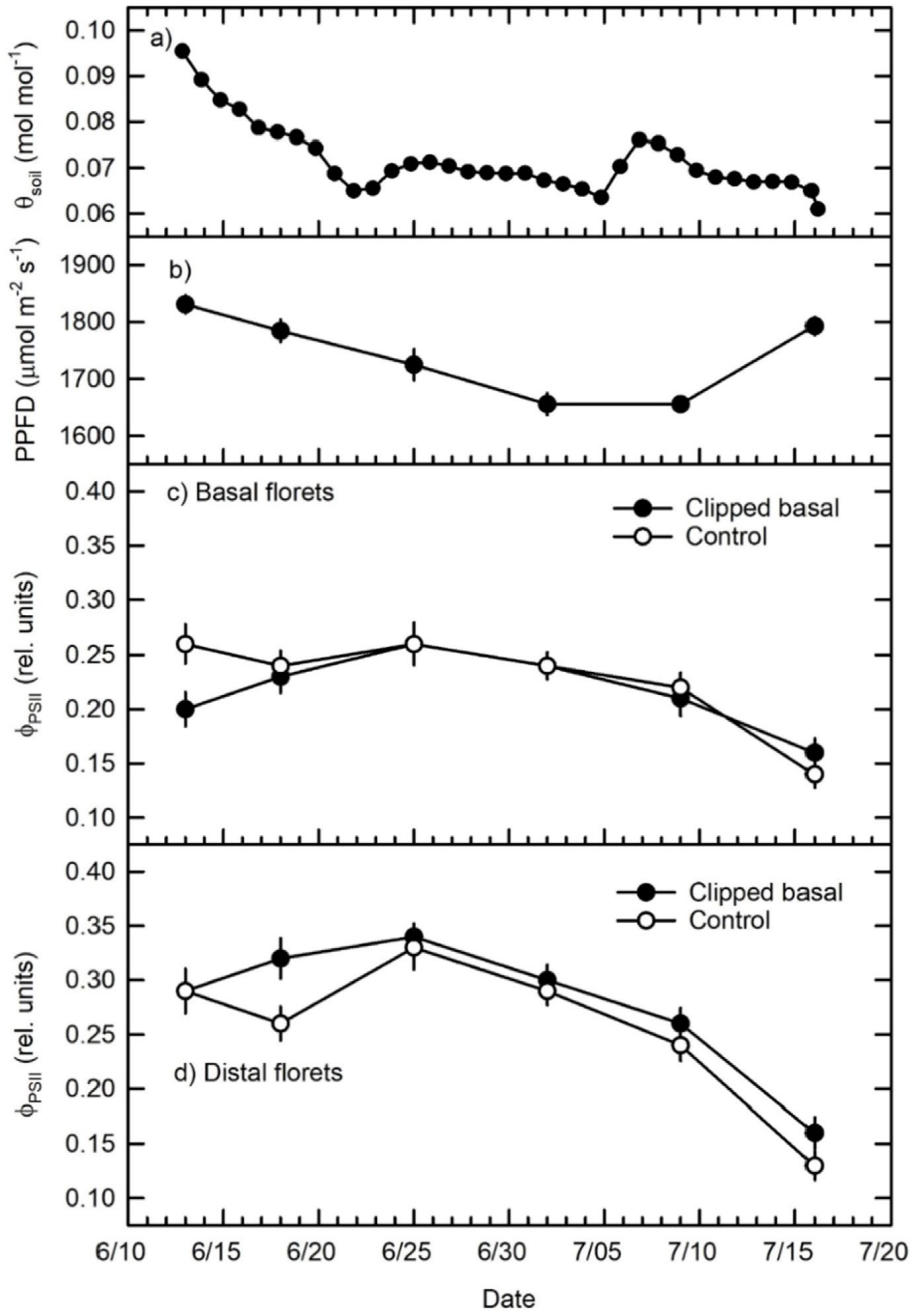
Reproductive period (a) volumetric soil moisture (*θ*_soil_) (b) photosynthetic photon flux density (PPFD) and light adapted PSII quantum efficiency (*ϕ*_PSII_) of (c) clipped and unclipped basal and (d) florets distal to these; PPFD and *ϕ*_PSII_ are means of 32 and 16 measurements, respectively; error bars indicate ± one SE of the mean. Statistical analyses for phenological stages are: Pre‐anthesis (measurements 1 and 2), anthesis (measurements 3 and 4), and post‐anthesis (measurements 5 and 6).

Volumetric soil moisture (*θ*_soil_) over the course of the study followed a typical seasonal drying trend, reducing from high levels attained over early to mid‐spring rainfall (Figure 1a). These *θ*_soil_ are very similar to those experienced in previous studies in this location (Hamerlynck and O'Connor 2022; Hamerlynck et al. 2023; Hamerlynck et al. 2024). Incident photosynthetic photon flux densities (PPFD; Figure 1b) were consistently above 1500 μmol m^−2^ s^−1^, well above documented photosynthetic light saturation points for crested wheatgrass seedheads (Hamerlynck and Ziegenhagen 2020). Two days following basal floret defoliation, *ϕ*_PSII_ in clipped florets declined below control floret levels, then recovered to control floret levels throughout the rest of the reproductive period (Figure 1c). Florets distal to clipped florets started at *ϕ*_PSII_ similar to controls, then attained higher *ϕ*_PSII_ even as levels declined through the rest of the study (Figure 1d). Despite the initial difference, basal floret *ϕ*_PSII_ did not differ significantly between clipped and unclipped controls pooled across the study but rather changed significantly between phenological stages (Table 1), being significantly lower over the post‐anthesis compared to similar higher *ϕ*_PSII_ over the pre‐anthesis and anthesis stages (Table 2). Basal clipping resulted in significant differences between distal floret *ϕ*_PSII_ (Table 1). This in part was due to higher *ϕ*_PSII_ in florets distal to clipped at the end of the pre‐anthesis, and *ϕ*_PSII_ in these florets being consistently higher than those above intact basal florets throughout the reproductive period (Figure 1d; Table 2), resulting in significantly higher *ϕ*_PSII_ in distal to clipped (0.28 ± 0.009 SE) compared to those distal to controls pooled across the study (0.26 ± 0.009 SE; LSD < 0.05). As in basal florets, distal floret *ϕ*_PSII_ were high and similar over the pre‐anthesis and anthesis stages and these were significantly higher than *ϕ*_PSII_ attained over the post‐anthesis (Figure 1d; Table 2).

**TABLE 1:** Repeated‐measures analysis of variance *F*‐test results comparing basal (direct) and distal (indirect) photochemical (*F*_v_/*F*_m_ and *ϕ*_PSII_) and non‐photochemical (*ϕ*_NPQ_ and *ϕ*_NO_) responses to basal floret clipping treatment over pre‐anthesis, anthesis, and post‐anthesis reproductive phenological stages. Degrees of freedom for each *F*‐test in parentheses, bold results significantly differ at *p* < 0.05.

Structure‐parameter	Basal clip_(1,30)_	Phenological stage_(2,60)_	Clip × stage_(2,60)_
Basal floret—*ϕ*_PSII_	1.44	**24.52**	2.21
Basal floret—*F*_v_/*F*_m_	**53.54**	0.62	**6.34**
Basal floret—*ϕ*_NPQ_	1.17	**3.86**	**6.33**
Basal—*ϕ*_NO_	0.14	1.49	1.15
Distal floret—*ϕ*_PSII_	**6.71**	**58.15**	0.25
Distal floret—*F*_v_/*F*_m_	1.98	**13.56**	1.53
Distal floret—*ϕ*_NPQ_	2.32	**11.28**	0.62
Distal floret—*ϕ*_NO_	**6.63**	**5.22**	1.00

**TABLE 2:** Means and standard errors of basal floret (direct) and distal floret (indirect) photochemical (*F*_v_/*F*_m_ and *ϕ*_PSII_) and non‐photochemical (*ϕ*_NPQ_ and *ϕ*_NO_) responses to basal floret clipping over pre‐anthesis, anthesis, and post‐anthesis reproductive phenological stages. Superscripted letters differ at *p* < 0.05 (LSD), bold results indicate significant clipping treatment differences within a phenological stage, italic and underlined results indicate significant differences between phenological stages within a clipping treatment.

Position	Pre‐Anthesis		Anthesis		Post‐Anthesis	
	Base clip	Control	Base clip	Control	Base clip	Control
Basal						
*ϕ*_PSII_	0.21 (0.011)^b^	0.25 (0.011)^a^	0.25 (0.011)^a^	0.25 (0.001)^a^	0.18 (0.011)^c^	0.18 (0.011)^c^
*F*_v_/*F*_m_	***0.65 (0.011)*** ^ ***c*** ^	***0.72 (0.005)*** ^ ***a*** ^	**0.65 (0.010)** ^**c**^	**0.70 (0.007)** ^**ab**^	*0.68 (0.012)* ^ *b* ^	0.69 (0.007)^b^
*ϕ*_NPQ_	***0.47 (0.038)*** ^ ***a*** ^	**0.34 (0.026)** ^**c**^	*0.38 (0.032)* ^ *bc* ^	0.41 (0.021)^ab^	*0.45 (0.026)* ^ *ab* ^	0.48 (0.021)^a^
*ϕ*_NO_	0.38 (0.045)^a^	0.43 (0.031)^a^	0.38 (0.033)^a^	0.34 (0.020)^a^	0.37 (0.025)^a^	0.34 (0.019)^a^
Distal						
*ϕ*_PSII_	0.30 (0.012)^ab^	0.28 (0.013)^b^	0.32 (0.010)^a^	0.31 (0.012)^a^	0.21 (0.014)^c^	0.18 (0.013)^c^
*F*_v_/*F*_m_	0.69 (0.006)^a^	0.69 (0.005)^a^	0.67 (0.006)^b^	0.66 (0.007)^bc^	0.67 (0.009)^b^	0.64 (0.013)^c^
*ϕ*_NPQ_	0.37 (0.020)^bc^	0.33 (0.028)^c^	0.40 (0.018)^bc^	0.40 (0.015)^bc^	0.48 (0.020)^a^	0.44 (0.031)^ab^
*ϕ*_NO_	**0.32 (0.021)** ^**bc**^	***0.39 (0.027)*** ^ ***a*** ^	0.28 (0.018)^c^	*0.29 (0.016)* ^ *c* ^	0.31 (0.019)^bc^	*0.38 (0.033)* ^ *ab* ^

**FIGURE 2:**
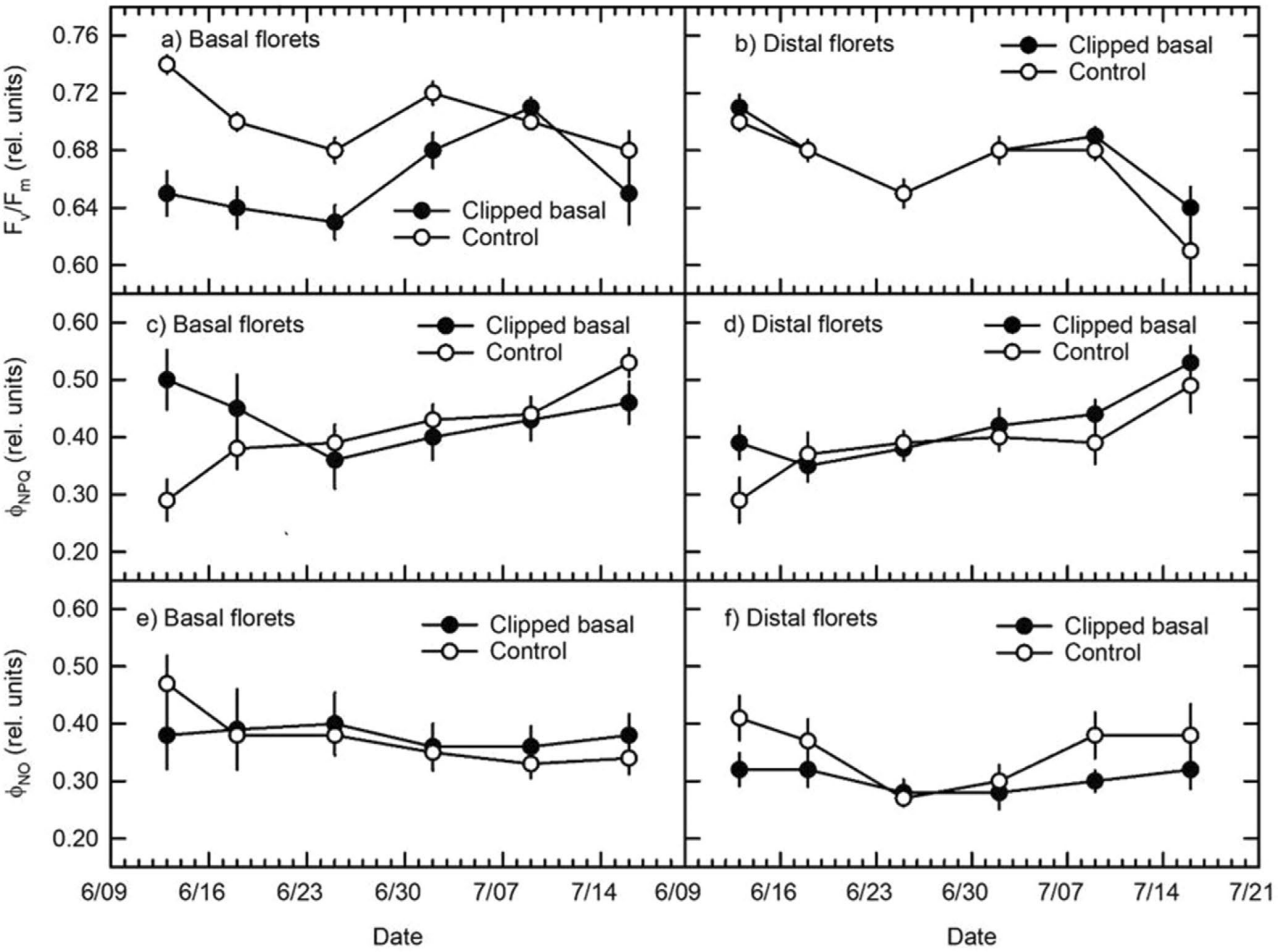
Reproductive period responses to basal floret clipping of basal florets (direct effect) and distal florets (indirect effect) optimal PSII quantum yield (*F*_v_/*F*_m_), light‐adapted PSII quantum yield (*ϕ*_PSII_), and quantum yield of regulated (*ϕ*_NPQ_) and unregulated (*ϕ*_NO_) non‐photochemical quenching. Symbols are the average 16 measurements; error bars indicate ± one SE of the mean. Statistical analyses for phenological stages are: Pre‐anthesis (measurements 1 and 2), anthesis (measurements 3 and 4), and post‐anthesis (measurements 5 and 6).

Optimal PSII quantum yield (*F*_v_/*F*_m_) in clipped basal florets was depressed below control floret levels over the first four samples, corresponding to pre‐anthesis and anthesis stages, followed by convergence to controls over the post‐anthesis (Figure 2a). Pre‐anthesis low *F*_v_/*F*_m_ was concurrent with increased *ϕ*_NPQ_ in clipped basal florets (Figure 2c), while *ϕ*_NO_ was at similar levels, as it was throughout the full study period (Figure 2e). Pooled across the study, there was a significant difference between clipped and unclipped basal floret *F*_v_/*F*_m_, with a significant stage‐by‐clip interaction (Table 1). Overall, clipped basal floret *F*_v_/*F*_m_ (0.66 ± 0.006 SE) was lower than in unclipped counterparts (0.70 ± 0.004 SE; LSD *p* < 0.05). The two‐way interaction was due to: (i) significantly lower clipped basal floret *F*_v_/*F*_m_ over the pre‐anthesis and anthesis stages, with significant differences over the post‐anthesis (Table 2) and (ii) increasing *F*_v_/*F*_m_ going from pre‐ to post‐anthesis in clipped florets while control floret *F*_v_/*F*_m_ was less variable across the three phenological stages (Table 2; Figure 2a). Seasonally pooled basal *ϕ*_NPQ_ significantly differed between treatments (0.43 ± 0.019 SE for clipped and 0.41 ± 0.014 SE for controls, respectively), also with a stage‐by‐clip interaction (Table 2). This was due to pre‐anthesis basal floret *ϕ*_NPQ_ being significantly higher than in control florets over the anthesis and post‐anthesis stages (Table 2) despite *ϕ*_NPQ_ levels being quite close between treatments by the end of the pre‐anthesis stage (Figure 2c). There were also within‐treatment stage differences, with clipped basal florets having high *ϕ*_NPQ_ during pre‐anthesis, followed by low levels during anthesis, then increasing again post‐anthesis, while control basal florets had a trend of increasing *ϕ*_NPQ_ going from pre‐ to post‐anthesis (Table 2; Figure 2c). There was no significant variation in basal floret *ϕ*_NO_ between clipping treatments or phenological stages across the reproductive period (Table 2; Figure 2e).

Unlike distal floret *ϕ*_PSII_, *F*_v_/*F*_m_ did not significantly differ between clipping treatments but only varied significantly between phenological stages (Table 1). Distal floret *F*_v_/*F*_m_ were high and similar over pre‐anthesis and anthesis stages, and these were significantly greater than *F*_v_/*F*_m_ attained post‐anthesis (Figure 2b, Table 2). *ϕ*_NPQ_ in distal florets were similar between basal clipping treatments and showed significant increases progressing from pre‐anthesis to post‐anthesis stages (Figure 2d; Table 2). In contrast to basal florets, *ϕ*_NO_ in distal florets differed significantly between basal floret clipping treatments (Table 1). Florets distal to clipped basal florets had significantly lower *ϕ*_NO_ (0.30 ± 0.011 SE) compared to those distal to controls (0.35 + 0.016 SE) pooled across the study. The higher *ϕ*_NO_ in distal to control florets apparent pre‐ and post‐anthesis (Figure 2f; Table 2) likely drove the significant differences in *ϕ*_NO_ between the phenological stages (Table 1).

## 4 Discussion

In both basal and distal florets, *F*_v_/*F*_m_ was below 0.75, a level indicative of the presence of priming and engagement of xanthophyll‐cycle mediated photoprotective thermal dissipation of excess absorbed PSII energy (Demmig‐Adams and Adams III 1992; Osmond 1994). Higher *ϕ*_NPQ_ and lower *F*_v_/*F*_m_ in clipped basal florets compared to those in unclipped controls are highly suggestive of high engagement of regulated photoprotective NPQ. These findings partially support our hypothesis that clipping would result in higher NPQ and down‐regulation of PSII quantum efficiency and suggest Hamerlynck et al. (2023) conjecture was correct, that floral defoliation induced down‐regulation of PSII photochemical function was mediated by non‐photochemical photoprotective mechanisms. However, contrary to our expectations and in contrast to findings by Hamerlynck et al. (2023) and Quigley et al. (2024), there was no evident down‐regulation of light‐adapted *ϕ*_PSII_ in clipped basal florets. This may be due to differences in actinic light sources; our current study assessed *ϕ*_PSII_ under full spectrum sunlight, while our previous results were obtained with an instrument using an artificial red/blue actinic light source for concurrent gas exchange and chlorophyll fluorescence measurements. Quantum yield can vary in response to both light quality and quantity (Hogewoning et al. 2012; Jokic et al. 2025), and the spectral differences in actinic light sources between our studies may have affected the attained *ϕ*_PSII_.

Lower *F*_v_/*F*_m_ and higher *ϕ*_NPQ_ (Figure 2a,c) in clipped basal florets were not always concurrent with decreased *ϕ*_PSII_; indeed, *ϕ*_PSII_ was generally similar between clipped and unclipped controls (Figure 1c; Table 2). *ϕ*_NPQ_ does not reflect the magnitude of NPQ engagement, but rather the proportion of absorbed light energy diverted safely away from PSII reaction centers, and *ϕ*_PSII_ is considered a more sensitive indicator of physiological stress than *F*_v_/*F*_m_ (Klughammer and Schreiber 2008). Lower *F*_v_/*F*_m_ in clipped florets suggests stronger engagement and slower reversing of regulated NPQ, possibly due to greater regulatory involvement by the thylakoid‐bound PsbS protein (Li et al. 2002). Regardless, the degree of NPQ engagement was probably not sustained enough to reduce subsequent light‐adapted *ϕ*_PSII_. In a compensatory context, maintaining *ϕ*_PSII_ makes sense, as electron transport rates would not be under additional photoprotective restraint, and up‐regulation of photosynthetic activity could be more readily attained. In addition, it may be that reproductive post‐defoliation physiological processes may not follow an obligate post‐defoliation dynamic and differ between species and environmental conditions. Hamerlynck et al. (2023) made measurements after an exceptionally large rain during the pre‐anthesis period, while Quigley et al. (2024) examined reproductive compensatory photosynthetic responses of bluebunch wheatgrass, which has distinctly different reproductive photosynthetic characteristics to crested wheatgrass (Hamerlynck et al. 2019; Hamerlynck and Ziegenhagen 2020). Therefore, interannual variation in reproductive period environmental conditions may affect the timing and magnitude of floral defoliation photosynthetic responses.

There were distinct indirect responses to clipping of basal florets apparent in the photochemical and non‐photochemical processes in florets distal to them. While *F*_v_/*F*_m_ and *ϕ*_NPQ_ did not differ in distal florets between clipping treatments, *ϕ*_PSII_ and *ϕ*_NO_ did, with higher overall *ϕ*_PSII_ and lower *ϕ*_NO_ in florets above clipped florets compared to those distal to controls (Figure 2 and Table 2). *ϕ*_NO_ reflects constitutive PSII non‐photochemical processes (Klughammer and Schreiber 2008). High light exposure increases the rate of photochemical reactions, increasing the reduction state of the photosynthetic electron transport chain, principally at the primary electron acceptor, Q_a_, though other electron transport molecules can be affected as well, such that decreases in *ϕ*_PSII_ are compensated for by an increase in *ϕ*_NO_ (Samson et al. 2019). We found clipping of basal florets induced relaxation of PSII redox conditions in their distal florets such that *ϕ*_PSII_ was maintained at modest but consistently higher levels than those distal to unclipped ssflorets (Figure 1d). Lower *ϕ*_NO_ and higher *ϕ*_PSII_ suggest smoother electron transfer through the entire photosynthetic electron transport system, but our results do not provide definitive insight as to the mechanism(s) by which this is attained. Photorespiration is an alternative electron transport system intimately linked to enzymatically mediated nitrogen dynamics and can affect gas exchange dynamics and NPQ, including *ϕ*_NO_ (Heber et al. 1996; El‐Khatib et al. 2004; Proctor and Smirnoff 2011; Zhong et al. 2022). Higher *ϕ*_NO_ expression over the pre‐ and post‐anthesis stages in florets where intra‐seed‐head competition between basal and distal florets is intact (Warringa et al. 1998) might reflect competition for assimilated nitrogen affecting photorespiratory processes. This is an attractive idea in that photorerespiratory metabolites associated with high NPQ capacity such as H_2_O_2_ can affect subsequent herbivore behavior (Jänkäpää et al. 2013). However, the accumulation of photorespiratory and other photoprotective metabolites are usually associated with increased *ϕ*_NO_ (Proctor and Smirnoff 2011; Zhong et al. 2022), not its relaxation, as expressed in florets distal to clipped basal florets.

Another possibility is that the relaxation of *ϕ*_NO_ is related to carbon assimilation. Hamerlynck et al. (2023) suggested carbon assimilates from damaged basal florets supplemented those assimilated by unaffected distal florets, thereby enhancing their reproductive effort. Over the pre‐anthesis stage this supplementation could help support a larger number of viable ovules, much as the flag leaf does in crested wheatgrass (Hamerlynck et al. 2024). This exogenous supply could reduce the endogenous demand of these, thereby relaxing redox pressures to electron transport. The *ϕ*_NO_ relaxation with higher *ϕ*_PSII_ over the post‐anthesis stage could also support higher net photosynthetic rates (A_net_) during energetic provisioning. Hamerlynck et al. (2023) observed slightly elevated post‐anthesis distal A_net_ but conjectured most of the carbon that increased distal seedhead specific mass came from damaged basal florets. While our results don't negate this supposition, they do suggest that photosynthetic activity by the distal florets themselves could be enhanced and contribute more to seed provisioning than previously thought.

In conclusion, this study highlights the importance of establishing reproductive ecophysiological processes, as these often serve as the origin point of population and community dynamics (Bazzaz 1979; Bazzaz et al. 1979). Moreover, these results are consistent with others showing drought and herbivory pressures produce co‐adaptive ecological strategies, especially in aridland bunchgrasses (Adler et al. 2004; Quiroga et al. 2010). This study and others we have made establish crested wheatgrass as possessing a remarkably integrated reproductive photosynthetic apparatus that facilitates its ability to produce seeds more capable of overcoming environmentally imposed demographic bottlenecks than native bunchgrasses (James et al. 2011; Hamerlynck and Davies 2019; Hamerlynck et al. 2019; Hamerlynck and O'Connor 2021; Hamerlynck et al. 2023; Quigley et al. 2023; Hamerlynck et al. 2024). Additionally, recent work has sought to formulate and implement seed‐based restoration strategies based on Grubb's regeneration niche concept (Grubb 1977; Larson et al. 2023, 2025). Our study suggests such approaches could be refined and improved with information regarding the environmental and biological constraints to parental‐plant reproductive ecophysiological performance.

## Funding

This work was supported by the Agricultural Research Service and Oregon State Agricultural Experiment Station.

## Conflicts of Interest

The authors declare no conflicts of interest.

## Data Availability

All data presented in this manuscript will be freely available at the USDA AgData Commons (https://agdatacommons.nal.usda.gov/; DOI: https://doi.org/10.15482/USDA.ADC/31100302).
